# A Fire Reconnaissance Robot Based on SLAM Position, Thermal Imaging Technologies, and AR Display

**DOI:** 10.3390/s19225036

**Published:** 2019-11-18

**Authors:** Sen Li, Chunyong Feng, Yunchen Niu, Long Shi, Zeqi Wu, Huaitao Song

**Affiliations:** 1 School of Building Environment Engineering, Zhengzhou University of Light Industry, 5 Dongfeng Road, Zhengzhou 450002, Henan, China; lisen@zzuli.edu.cn (S.L.); fcyong@zzuli.edu.cn (C.F.); niuyunchen@zzuli.edu.cn (Y.N.); wu@zzuli.edu.cn (Z.W.); songhuaitao@zzuli.edu.cn (H.S.); 2 Civil and Infrastructure Engineering Discipline, School of Engineering, RMIT University, Melbourne, VIC 3000, Australia

**Keywords:** fire reconnaissance robot, SLAM, thermal imaging, AR

## Abstract

Due to hot toxic smoke and unknown risks under fire conditions, detection and relevant reconnaissance are significant in avoiding casualties. A fire reconnaissance robot was therefore developed to assist in the problem by offering important fire information to fire fighters. The robot consists of three main systems, a display operating system, video surveillance, and mapping and positioning navigation. Augmented reality (AR) goggle technology with a display operating system was also developed to free fire fighters’ hands, which enables them to focus on rescuing processes and not system operation. Considering smoke disturbance, a thermal imaging video surveillance system was included to extract information from the complicated fire conditions. Meanwhile, a simultaneous localization and mapping (SLAM) technology was adopted to build the map, together with the help of a mapping and positioning navigation system. This can provide a real-time map under the rapidly changing fire conditions to guide the fire fighters to the fire sources or the trapped occupants. Based on our experiments, it was found that all the tested system components work quite well under the fire conditions, while the video surveillance system produces clear images under dense smoke and a high-temperature environment; SLAM shows a high accuracy with an average error of less than 3.43%; the positioning accuracy error is 0.31 m; and the maximum error for the navigation system is 3.48%. The developed fire reconnaissance robot can provide a practically important platform to improve fire rescue efficiency to reduce the fire casualties of fire fighters.

## 1. Introduction

Fire casualties occur not only for occupants but also for firefighters. Recently, many firefighters have lost their lives during fire rescuing processes. A statistical study [1] indicated that 742 firefighters were killed in the U.S. during 2006–2014, while the average deaths were 73 during 2011– 2016 [2]. One of the main reasons is because of the unknown and hazardous fire environment with toxic gases, high temperature, dense smoke, and low amount of oxygen [3,4].

One of the major challenges for fire rescue is detection, such as fire location, temperature and smoke density, and the available route. The accuracy of the detection not only determines the selection of rescue plan but also the result of the selected rescue plan [5]. It is crucial for the firefighters to clearly understand the relevant information under complicated fire conditions before entering the building. Normally, fire detection includes external inspection, consulting the evacuees from the fire, and internal detection. The external inspection is very much based on the experience of firefighters but they usually can only obtain limited information, such as based on flame type, direction, the color and smell of smoke, and movement characteristics. Consultation with the escapers from a fire usually lacks accuracy as the evacuees are quite stressful after escaping from the fire and the fire scenarios are also changing all the time. 

Among these three methods, internal detection may be currently the most direct one for firefighters. Before entering the building, firefighters should be equipped with a breathing device, oriented rope, high-power flashlight, etc. However, the acquirement of the information is limited by the carried oxygen cylinder and flashlight. Moreover, the firefighters are always under high risk. Based on the above analysis, it is critically important to develop a fire reconnaissance robot to overcome the problems mentioned above.

**Figure 1:**
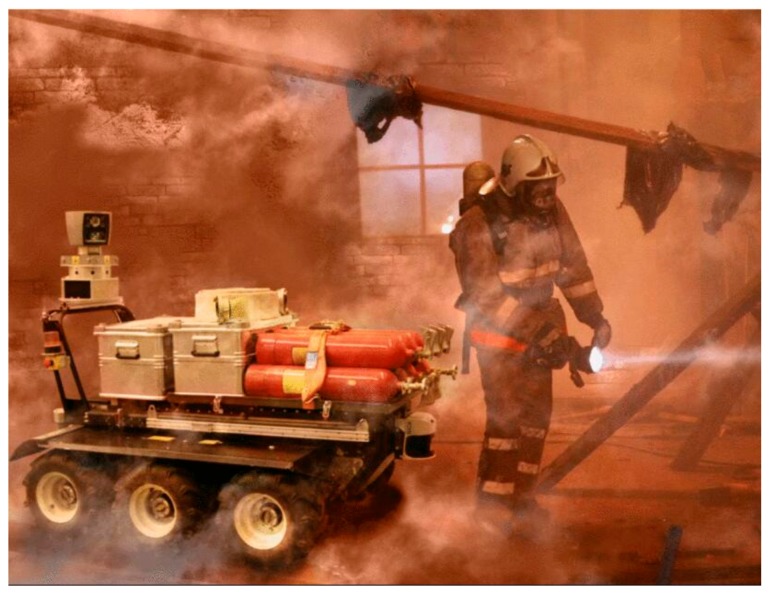
Firefighter follower robot called Longcross.

A large number of studies have been carried out regarding the development of a fire robot. Based on their equipped devices and functions, these robots can be further divided into four types, namely auxiliary rescue robot, inspection robot, fire suppression robot, and reconnaissance robot. An auxiliary rescue robot is designed to assistant firefighters regarding their walking, transport of devices, and fire suppression. For example, Li et al. [6] developed a walking assist device based on a modified ultrasonic obstacle avoidance technology. Figure 1 shows a robot, called Longcross [7], which can follow the firefighters and offer assistance under fire conditions. It can help to transport occupants and devices under emergency conditions, and also suppress fire together with the firefighters. Another example is Octavia [8], which is a humanoid robot that is equipped with a fire extinguisher. It can suppress fire together with firefighters and also clear obstacles with control of gesture and voice.

The inspection robot aims to inspect the specified areas under fire conditions, which can also suppress the fire independently. For example, Sucuoglu et al. [9] designed an obstacle avoidance robot equipped with smoke, temperature and flame sensors, which can detect the fire within 1 m with an accuracy of 92%. SAFFiR [10] is a humanoid firefighting robot, which can avoid obstacles on a ship, maintain balance, and walk upright. It can control a fire extinguisher even though it is not directly equipped with firefighting equipment. Alhaza et al. [11] designed an indoor firefighting robot equipped with fire extinguisher. It can detect the fire source based on flame detection technology and firefighters can confirm the fire from a camera and then take the relevant actions. Unmanned aerial vehicle (UAV) is another type of fire inspection. It shows excellent maneuverability and a broad working area, which can be adopted for aerial inspection and suppression of early-stage fire [12].

**Figure 2:**
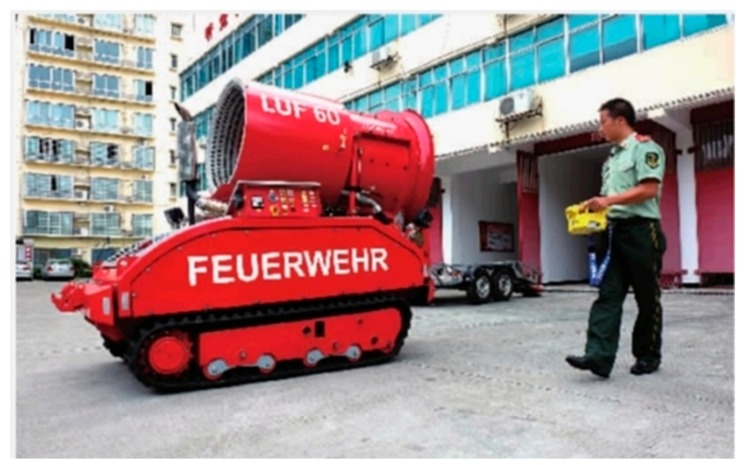
A firefighting robot called LUF60.

A fire suppression robot is used to suppress the fire through remote control of the firefighters. For example, Zhang and Dai [13] designed a remotely controllable robot that is able to replace firefighters in the hazardous areas for rescuing processes. Another robot, called JMX-LT50 [14], is equipped with a high-pressure water jet, where a camera on it is used to assist the remote control of a water jet. Figure 2 shows a firefighting robot, called LUF60 [15]. The high-power nozzle equipped in LUF60 can spray water in the form of water mist that can reduce the ambient temperature in a short time, move the toxic gases away, change the direction of smoke movement, and broaden the view during the rescuing process.

A reconnaissance robot is a type of robot mainly focusing on fire inspection. Its function is on passing important fire information to fire fighters but not direct fire suppression. For example, Kim et al. [16] developed an intelligent firefighting mobile robot. The robot is equipped with an obstacle avoidance function, which can find the fire source and plan the path to it through an infrared camera. Berrabah [17] designed a reconnaissance robot for the outdoor environment, which is equipped with GPS, inertial navigation, cameras, ultrasonic sensors, and various chemical sensors while the obtained information can be transferred to the control center for decision making.

Although many types of robots have been introduced above, the development of a firefighting robot is still at its starting stage. Several challenges and shortcomings still exist: (a) most of the firefighting robots focus on fire suppression, ignoring the detection in the surrounding environment. So their volume is quite big, which limits their mobility and applicability; (b) The function of mapping construction for the robots is hampered under the fire conditions. Although they are equipped with the original map of the building, they lack real-time mapping construction as the map could be changed quite significantly under fire conditions; (c) The controller of the robots is quite heavy so that it is inconvenient to carry. Therefore, the robot needs to be standing still to enable the relevant operation. It cannot free the hands of the firefighters, which limits the capability of their rescuing tasks, as shown in Figure 2.

Therefore, to overcome the above challenges, a small and flexible robot was developed for fire reconnaissance in this study, with the functions of operation display, map construction, and video monitoring. It enables the monitoring and operation of the robot based on AR technology, real-time map construction under fire conditions based on SLAM technology, and fire detection and rescuing based on the infrared thermos image technology and video surveillance system.

## 2. Description of the Robot System

**Figure 3:**
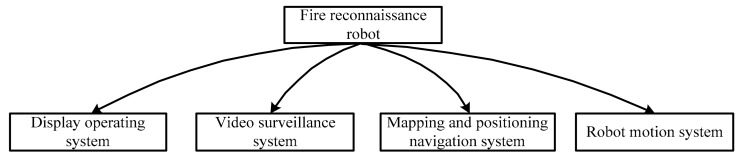
The structure of the developed fire reconnaissance robot.

For a robot dedicated to environmental reconnaissance, the video surveillance system is an indispensable part that can provide extensive information through images. Considering the complexity of the indoor environment, the mapping and positioning navigation system can then offer important support to firefighters to quickly reach the target position. Also, the display operation equipment carried by firefighters is an important part of a fire reconnaissance robot. Therefore, based on the above considerations, the developed fire reconnaissance robot in this study contains several systems, including a display operating system, a video surveillance system, a mapping, and positioning navigation system, and a motion system. The detailed structure of the developed robot can be seen in Figure 3.

### 2.1. Display Operation System

**Figure 4:**
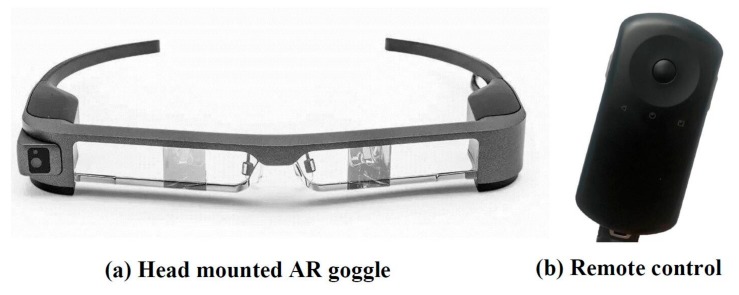
EPSON BT300.

To free the hands of the firefighters, the display operation system was developed based on the currently most advanced AR goggle technology (e.g., EPSON BT300, as shown in Figure 4). The goggle uses two 4.3-inch high-definition silicon OLEDs as the projection unit, which is equivalent to an 80-inch screen displayed in front of the firefighters. The resolution is as high as 1280 × 720, which can provide very comprehensive and clear images.

EPSON BT300 (Epson (China) Co., Ltd. Beijing, China) is equipped with remote control, as shown in Figure 4b. It is equipped with an Android 5.1 processing system, Intel Atom x 5 CPU, 2 GB RAM, and 48 G memory. It also adopts a touch sensor so that the control can be based on inputs such as touch and drag.

### 2.2. Video Surveillance System

**Figure 5:**
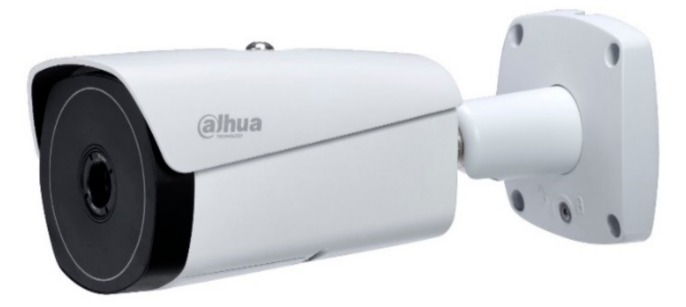
Thermal imaging camera DH-TPC-BF5400.

When compared with other scenarios, the requirements of the video surveillance system for fire conditions is much higher. Due to the attenuation of smoke on the light, ordinary cameras cannot be applied under fire conditions. To solve the problem, a thermal imaging camera is usually adopted to construct the video surveillance system. In this study, the thermal imaging camera was supplied by Zhejiang Dahua Technology in China (DH-TPC-BF5400), as shown in Figure 5. The system uses uncooled Vox infrared focal plane array, where the thermal imaging detector pixel is 400 × 300, and the imaging resolution is as high as 1280 × 1024. The system shows the advantages of low energy consumption, which enable 24 h of uninterrupted working.

### 2.3. Mapping and Positioning Navigation System

The mapping and positioning are based on technologies, such as AutoCAD map, ultra-wide band (UWB), wireless local area network (WLAN), Bluetooth, radio frequency identification (RFID), and ZigBee [18]. However, under fire conditions, the original map may be not applicable due to the collapse of walls or structural components under fire conditions. At the same time, the base station for the systems may encounter power failure and is not able to offer continuing support [19,20]. Therefore, a real-time mapping and positioning navigation system may be the best solution currently under fire conditions.

Based on the above analysis, a mapping and positioning navigation system was developed based on a SLAM technology. The advantage is that the map can be built in real time that does not rely on AutoCAD maps. There is also no need to pre-arrange additional infrastructure such as the base station. It can be seen from Table 1 that the SLAM technology does not require support from base station and shows high positioning accuracy. Due to the above-mentioned advantages, the developed system is suitable for the fire environment.

**Figure 6:**
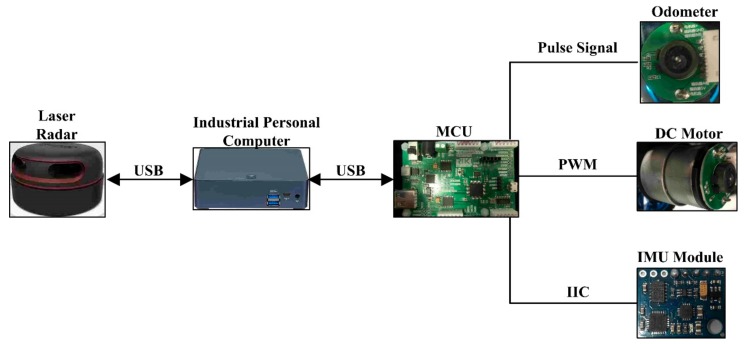
Hardware for the positioning and navigation of SLAM (MCU (microcontroller unit); DC Motor (Direct-Current Motor); IMU (Inertial Measurement Unit)).

Laser SLAM technology refers to a technique where the robot uses its own sensor to realize positioning during movement in an unexplored environment, and to inspect the surrounding environment to construct an incremental mapping to realize the positioning and navigation. The related hardware can be seen in Figure 6. Light detection and ranging (LiDAR, RPLIDAR-A2, SLAMTEC, shanghai, China) was adopted as the core detection unit to scan the surrounding area based on laser triangulation technology and to detect 2D point cloud information at the surfaces of the surrounding obstacles. The related information is then used to construct the map in an industrial personal computer (IPC) based on the SLAM algorithm. The technical parameters for the LiDAR are listed in Table 2.

Inertial measurement unit (IMU, GY-85, ADI, Norwood, America) was also adopted to collect the gesture information, such as walking speed and yaw. Also, an odometer was used to obtain the distance information of the walking robot. A microcontroller unit (MCU, STM32F103, STMicroelectronics, Geneva, Switzerland) was adopted to transfer the information to the IPC for post-processing. The movement of the robot is then realized by the DC motor driven by the MCU.

The robot has a problem in that the data collected by the sensor includes data of noise and measurement failure in the process of map construction. The problem will have a great impact on the accuracy of map construction because this system has uncertainty and external interference [21]. To solve this problem, Du et al used Kalman filters (KFs) and particle filters (PFs) to fuse the sensor data, thereby improving the accuracy and reliability of the system [22,23]. Therefore, in this research, the motion system of the robot is modeled by analyzing the data of the odometer, this model is regarded as the nonlinear model of the extended KFs. Then, the collected data of the odometer, IMU and LIDAR are respectively taken as the observation quantity and observation covariance matrix for status update, so as to obtain the updated system state and system covariance matrix. Finally, the updated state quantity and covariance matrix are the attitude information of the robot itself. The current position of the robot is estimated through the PFs to determine the position of the robot in the map.

### 2.4. The Overall Fire Reconnaissance Robot System

**Figure 7:**
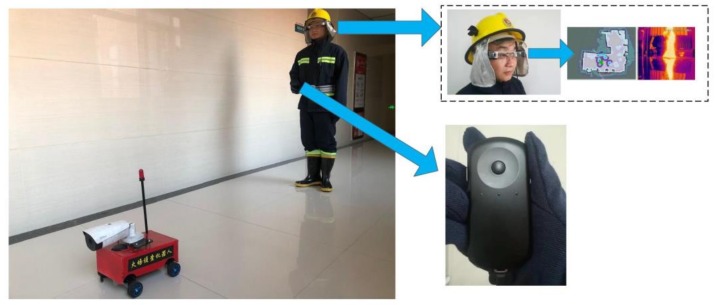
The operation of the developed fire reconnaissance robot.

**Figure 8:**
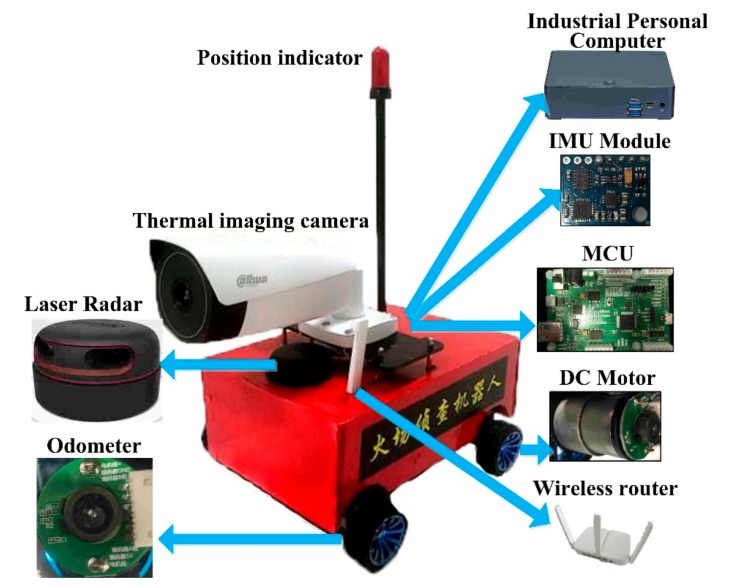
The structure of the developed fire reconnaissance robot (MCU (microcontroller unit); DC Motor (Direct-Current Motor); IMU (Inertial Measurement Unit)).

For the overall operation of the developed fire reconnaissance robot through this study please refer to Figure 7. Under fire conditions, the firefighters with AR goggles can follow the robot, while the firefighters do not need to carry anything as all the hardware can be placed on the robot, as shown in Figure 8. The robot is equipped with a router, and AR goggle and can communicate with the robot through a WIFI signal to acquire information such as video and map. Based on the communication, the robot can be controlled when it is within 50 m. The dimension of the robot is 30 cm (L) × 29 cm (W) × 30 cm (H), which can be driven by four 12 V DC motors. It has the advantages of small volume, excellent mobility, and applicability.

## 3. Performance Test of the Video Surveillance System

**Figure 9:**
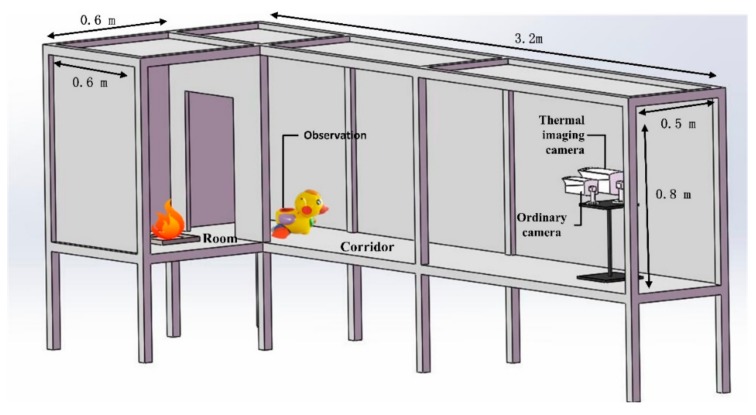
Experimental model to simulate a typical fire scenario with both room and corridor.

To address the applicability of the thermal imaging camera under fire conditions, an experimental model was developed to simulate the typical fire scenario, as shown in Figure 9. The experimental model includes a room connected to a corridor, while the fire source is located inside the room. The dimension of the room is 0.6 m × 0.6 m × 0.8 m (H), while for the corridor it is 3.2 m (L) × 0.5 m (W) × 0.8 m (H). The produced smoke inside the room can spread to the corridor through the door between them.

To test the performance of the video surveillance system, a color duck was adopted. Experimental results between the thermal imaging camera and ordinary camera were also compared. The ordinary camera was purchased from the same company with a thermal imaging camera, namely Zhejiang Dahua Technology in China. The ordinary camera is equipped with both visible and infrared imaging functions, where the infrared function can be automatically turned on when the ambient environment is dark. The fire source adopted typically n-heptane together with a smoke cake with ammonium, where the burning of the n-heptane and smoke cake can release a large amount of heat and smoke, respectively, to simulate the real fire conditions. The advantage of the combination is that it can quickly form an experimental environment with high temperature and dense smoke.

**Figure 10:**
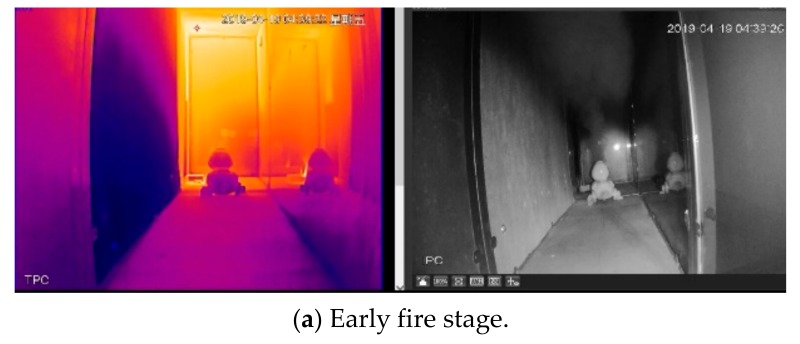

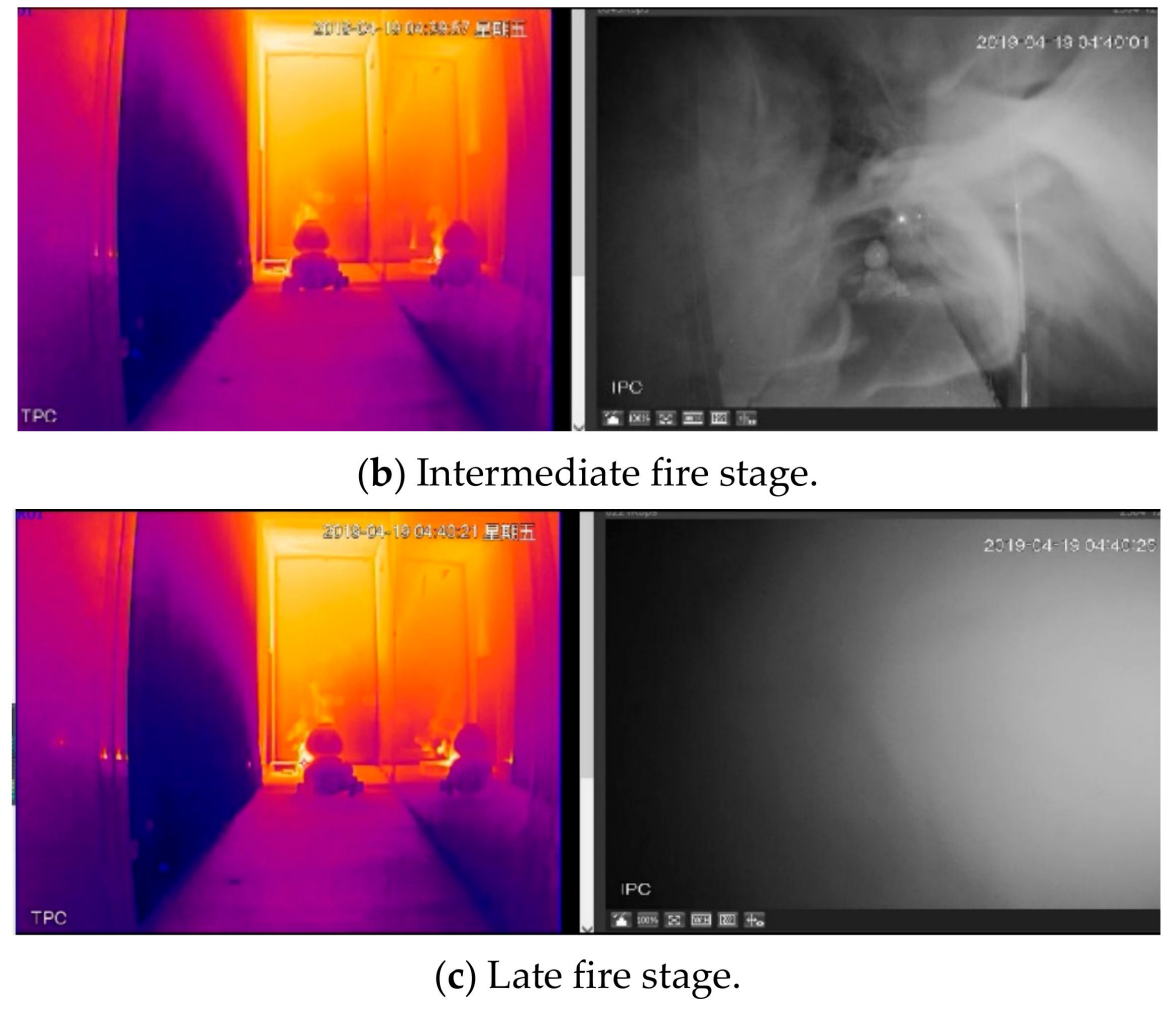
Comparisons between the obtained figures of thermal imaging and ordinary cameras under different fire stages.

The experimental results are shown in Figure 10, while the left and right figures show the outputs from thermal imaging and ordinary cameras, respectively. After the ignition of the fire, the ordinary camera changes to the infrared mode automatically when the surrounding environment is getting dark. This is the reason why the ordinary camera shows a gray-scale image. At the beginning of the test, the areas on both sides can be reflected by the ordinary camera. However, after the smoke becomes dense, the ordinary camera is unable to provide a clear image anymore. For the thermal imaging camera, the figures stay quite clear all the time, no matter which fire stage it is. The smoke shows a limited influence on the performance of the thermal imaging camera, as shown in Figure 10b. Even after more smoke is released, the thermal imaging camera is still capable of offering a clear figure, as shown in Figure 10c). Therefore, the viability of the adopted thermal imaging camera for fire conditions can be confirmed through experimental test. It can be considered as one of the feasible solutions for the current situation.

## 4. Performance Test of the Mapping and Positioning Navigation System

### 4.1. The Accuracy of Mapping Construction

**Figure 11:**
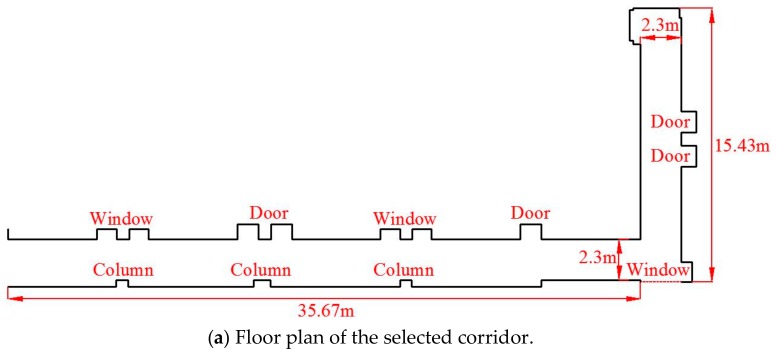

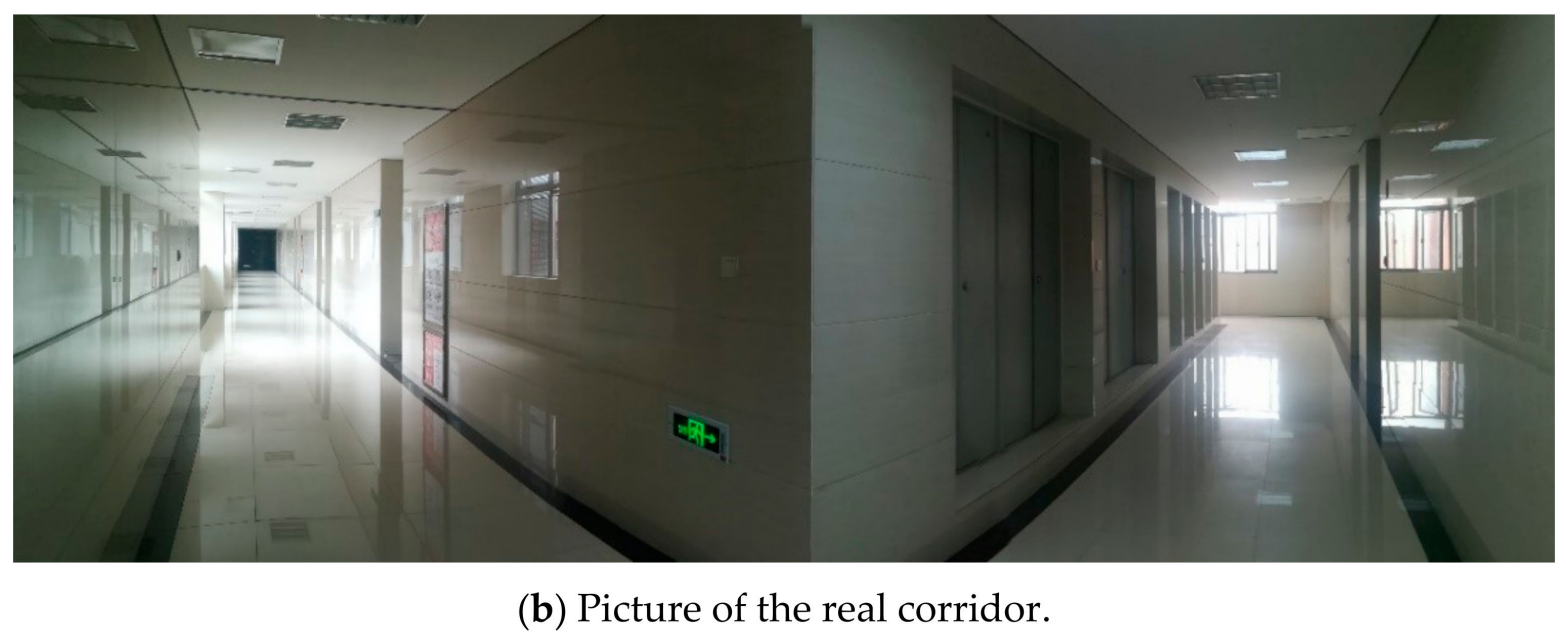
Selected L-shaped corridor for the experimental tests.

To validate the accuracy and reliability of the mapping and positioning navigation system of the developed fire reconnaissance robot, an L-shaped corridor was selected for the related experimental tests. The width and length of the selected corridor are 2.3 m and 51.1 m, respectively; while the long and short sides of the corridor are 35.67 m and 15.43 m, respectively. For the dimension and structure of this corridor refer to Figure 11. It can be seen from this figure that the doors and windows are convex along the corridor, which is quite challenging for the construction of mapping.

**Figure 12:**
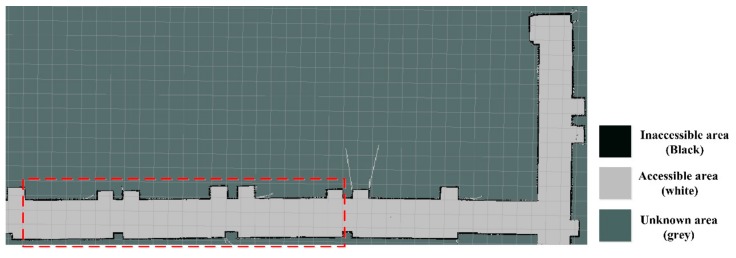
Occupancy grid mapping constructed by laser SLAM technology.

An occupancy grid mapping was built based on the laser SLAM technology, as shown in Figure 12, where various areas are different in color, such as black, white, and grey. It can be seen based on its comparison to the real corridor that the developed map can construct clearly not only the overall contour but featured structures, such as protruding columns, convex doors, and windows.

**Figure 13:**
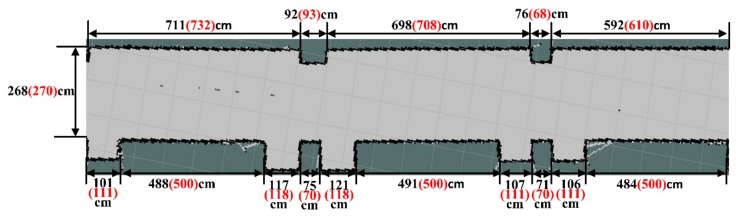
Constructed map with dimensions based on laser SLAM technology.

To further determine the accuracy of the constructed map, the area with a red dotted line in Figure 12 was extracted for detailed analysis. According to the scale of the constructed map, the actually represented size from each grid in the map can be calculated. For the obtained calculation results refer to Figure 13, while the values outside and inside the brackets represent the predicted and actual size. It can be seen that these two agree reasonably well.

**Figure 14:**
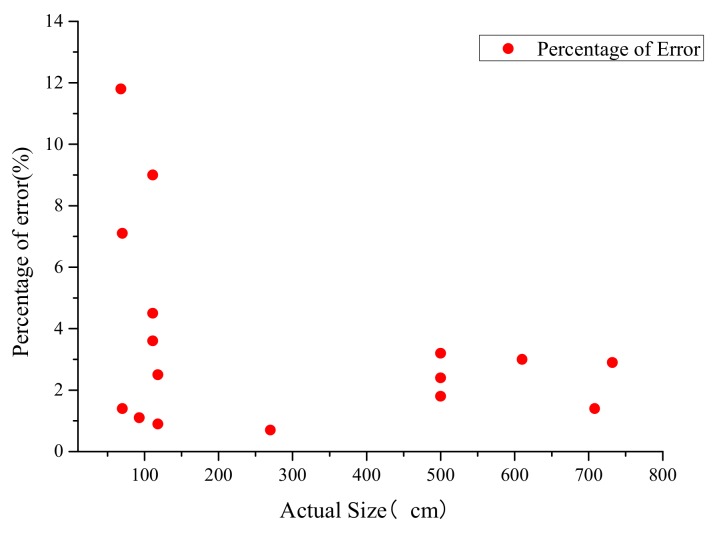
Differences between the predicted and actual dimensions of the corridor.

It can be seen from Figure 13 that the predicted and actual dimensions agree reasonably well. The relevant errors between these two groups can be seen in Figure 14. It can be seen from this figure that the maximum difference is 11.8%, while only three predictions are with higher than 5% differences. The average difference between these two groups of data is about 3.43%. Therefore, the accuracy and reliability of the laser SLAM technology can be confirmed from the experimental tests.

### 4.2. Accuracy Test of the Indoor Positioning

**Figure 15:**
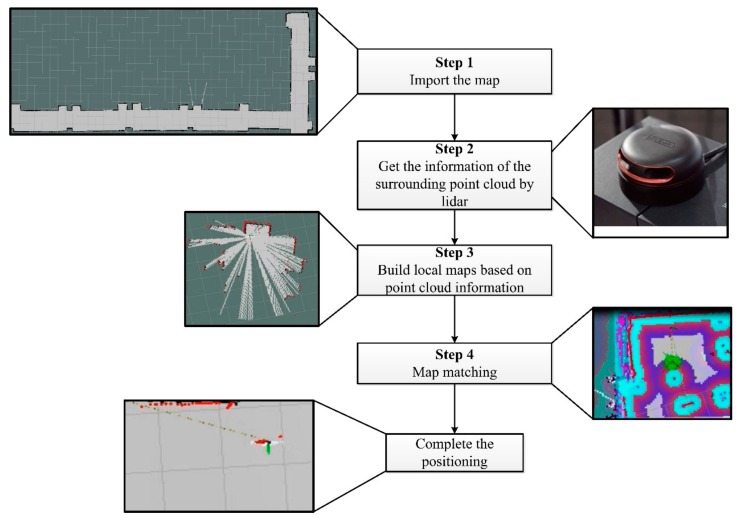
Flowchart of realizing the indoor positioning based on SLAM technology.

The flowchart for realizing the indoor positioning based on the SLAM technology can be seen in Figure 15. First, it needs to import the constructed occupancy grid mapping and confirm the initial attitude and position of the robot by acquiring information from the IMU module, odometer, and LiDAR. Then the 2D point cloud information within 12 m from the robot are obtained based on the scanning of the LiDAR. According to the 2D point cloud information, an area with a 6 m diameter can be scanned and a cost map is then constructed. The cost map is then compared with the actual floor plan to identify the location of the fire reconnaissance robot. This is the method for indoor positioning.

**Figure 16:**
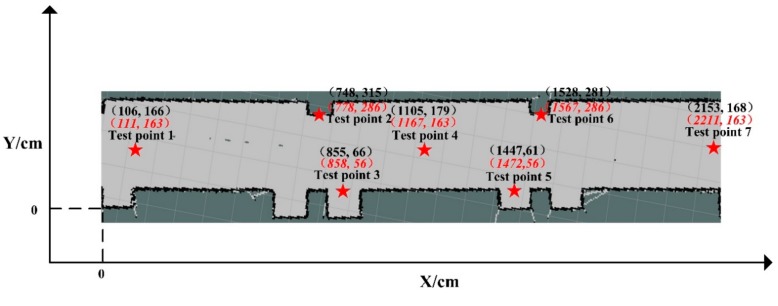
The predicted and actual coordinates of the robot.

A coordinate can be built for the selected corridor in Figure 11 when we set the lower-left corner of the map as the origin. The coordinate is shown in Figure 16. Seven locations in this map were selected to test the positioning accuracy of the robot, marked by pentagrams. The values in red italic and black are the predicted and actual coordinates of the map, respectively.

The positioning accuracy of the seven selected points can be calculated by,

(1) $$E=\frac{1}{N}\sum_{i=1}^{N}\sqrt{{\left(x_{i}^{p}-x_{i}^{b}\right)}^{2}+{\left(y_{i}^{p}-y_{i}^{b}\right)}^{2}}$$

where *E* is the positioning accuracy; *N* is the number of the tested points; $\left(x_{i}^{p},y_{i}^{p}\right)$ represents the predicted coordinate of the *i*^th^ point in the map; and $\left(x_{i}^{b},y_{i}^{b}\right)$ represents the actual coordinate of the *i*^th^ point in the actual corridor.

Based on the seven selected points, the average positioning accuracy can be obtained as 0.31 m. When comparing to the previous studies listed in Table 3, it can be seen that the obtained positioning accuracy from this study is ranked at the top when compared with the previous technologies.

### 4.3. The Accuracy of Navigation

Navigation is the process of planning a safe, fast, and collision-free path that can lead people to the terminal. The resulted path could determine the success of the navigation. In this study, the global A* algorithm, together with local D* algorithm, were adopted to plan the path. Through the global A* algorithm, the shortest path is confirmed by a global screening of all the possible nodes of the static data (offline data). As it adopts offline data, it is unable to handle the emergency events, such as suddenly appearing obstacles. D* is a heuristic searching algorithm based on a heuristic function that starts from the target node and combines sensors to traverse the nodes on the map towards the initial position. It is helpful to find the initial position to fully evaluate all nodes. Therefore, the D* algorithm can handle emergency events under fire conditions. However, it is unable to undertake an overall evaluation of all the possible nodes, which is used for the local path planning. It is then important to combine these two algorithms to promote their merits for the applications.

**Figure 17:**
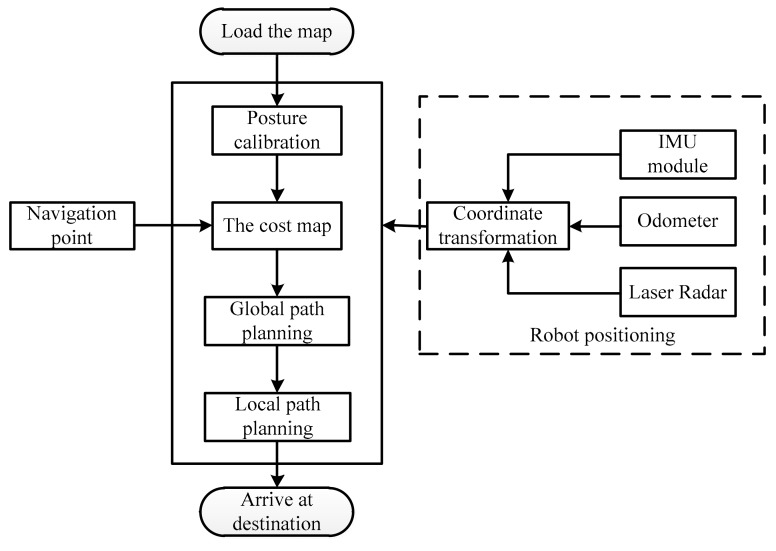
The navigation principle of the developed fire reconnaissance robot.

**Figure 18:**
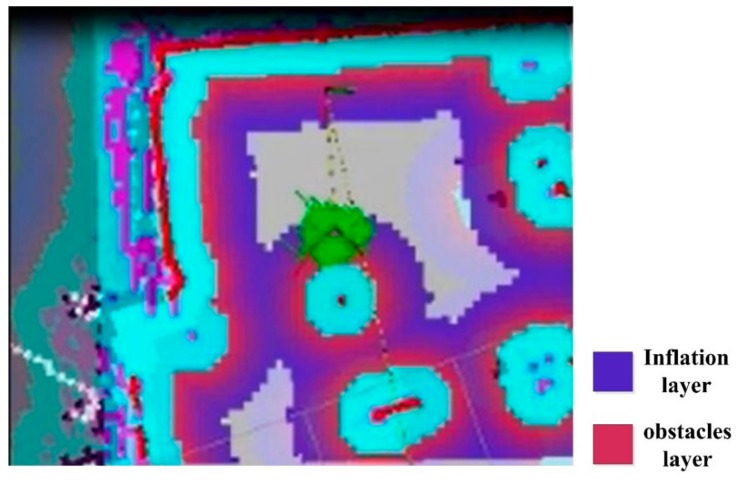
Inflation layer and obstacle layer in the cost map.

The navigation principle of the robot is shown in Figure 17. First, the occupancy grid mapping is loaded, while an attitude calibration is taken based on the IMU module, odometer, and LiDAR. The loaded occupancy grid mapping is a gray-scale map. However, the path planning in the static map does not consider the edges of the robot, which could hamper its safe walking. When there is no new obstacle appearing on the map, the map will not be updated dynamically. A cost map considering an expanded edge is then built before planning the path, as shown in Figure 18. In this figure, the blue part presents the inflation layer of the obstacles that the robot center cannot reach; and the red parts are the obstacles in the cost map that the edges of the robot cannot reach. In the cost map, the shortest path is obtained through the A* global path planning that can evaluate all the possible paths by screening all the nodes in the map. The robot is then navigated according to the global path. If there is any obstacle showing during the route, the D* local path planning will then be triggered to refine the path automatically by updating the cost map according to the obstacles.

To verify the reliability and accuracy of the navigation system, the navigation tests with and without any obstacle were undertaken accordingly. During the obstacle-free test, no obstacle was positioned inside the navigation environment. The purpose was to verify the accuracy of the system in the environment without any obstacle. During the obstacle test, obstacles were set inside the navigation environment on purpose to test its applicability inside a dynamically changing environment. This wais to make sure that the robot still performs navigation under emergency events.

#### 4.3.1. Obstacle-Free Navigation

**Figure 19:**
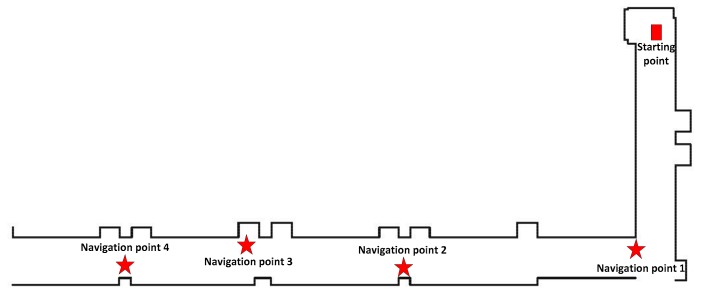
The floor plan of the obstacle-free navigation test.

In the experimental test, four navigation points were set for different purposes, as shown in Figure 19. The starting point was located 1.1 m from the end wall. Navigation point 1 was set after the first corner as a challenge, located 12 m away from the starting point. Navigation point 2 was located outside a column 27.3 m away from the starting point, where the robot could easily collide with the column if the navigation was not accurate. The situation for navigation point 3 was more complicated than the others in that the robot could be trapped inside the convex door when the navigation precision is challenging. Navigation point 4 was similar to point 2, but with a longer distance from the starting point.

**Figure 20:**
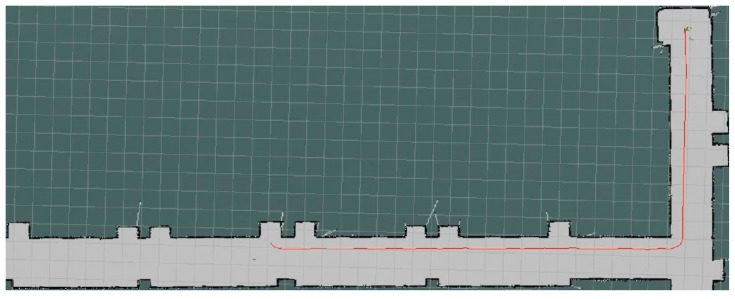
The path based on the global path planning for navigation point 3.

After the determination of the navigation point, the A* algorithm is then adopted to calculate the global path, as shown by the red lines in Figure 20. During the transport process of the robot, D* local path planning is also turned on to handle the emergency events, such as unexpected obstacles. For the obstacle-free test, the planned path from both A* and D* algorithms are the same when no obstacle is shown inside the navigation environment.

**Figure 21:**
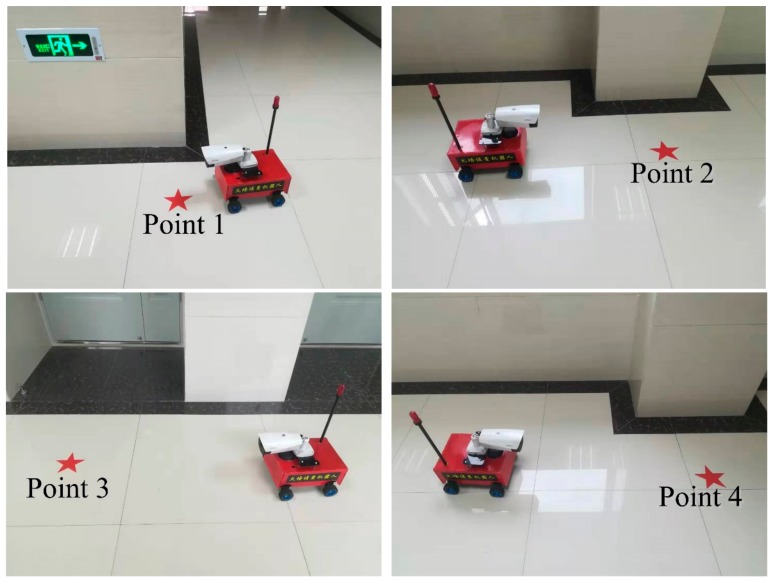
The differences between the navigated and actually arrived locations. The pentagrams are the navigated points, where the robot shows the actually arrived location.

According to the above-mentioned navigation points, the actual navigated locations of these four scenarios can be seen in Figure 21. It can be seen that the level of the differences is shown between the navigated and actually arrived locations.

**Figure 22:**
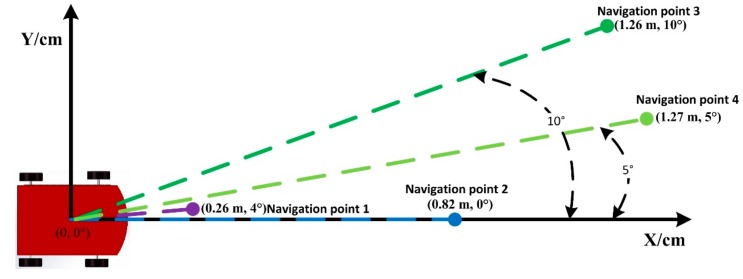
Distance and angle errors between the navigated and actually arrived points.

To analyze the navigation deviation, a coordinate was built, where the starting point of the robot is the origin, and the direction of traveling is the X-axis. The errors of the traveling distance and angle can be seen in Figure 22, and for the detailed values refer to Table 4. It can be observed that the distance error keeps increasing with a longer traveling distance, while the maximum occurs at the navigation point 4, namely 1.27 m. The minimum error occurs at navigation point 1, which is 0.26 m. This is due to the slippery floor in that the robot cannot stop immediately even after the stopping signal is already given. The trend for the angle error does not follow the same process of the distance. It can be seen that the maximum angle error occurs at the navigation point 3, namely 10°. The minimum error occurs at navigation point 2, where the error is 0°. The difference is also due to the slippery floor. Due to the uncertainty of the rotation, no clear relationship was found between the angle error and travelling distance.

#### 4.3.2. Navigation Test under Obstacle Environment

**Figure 23:**
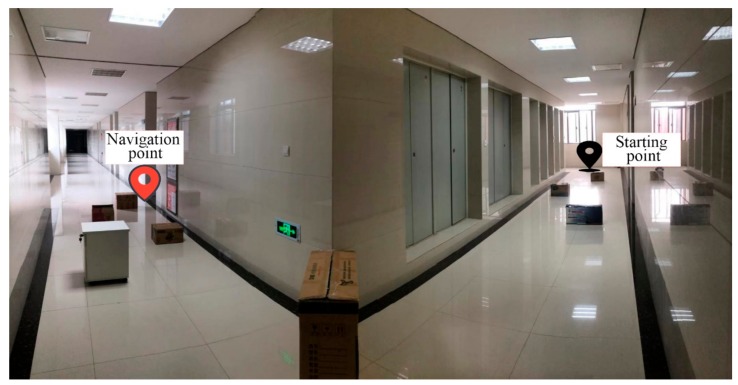
Navigation test with obstacles and the location of the eight obstacles.

The navigation test under an obstacle environment was undertaken to verify the applicability of the system in the dynamic environment and the response-ability under emergency events, respectively. A total of eight obstacles were positioned in the selected corridor, as shown in Figure 23. The distances of these eight obstacles from the starting points were 3.1 m, 5.7 m, 8.2 m, 12.9 m, 15.6 m, 16.8 m, 17.1 m, and 19.4 m, respectively. The starting point was 1.1 m away from the end wall. The navigation point was located inside the convex door, which was 23.6 m from the starting point.

**Figure 24:**
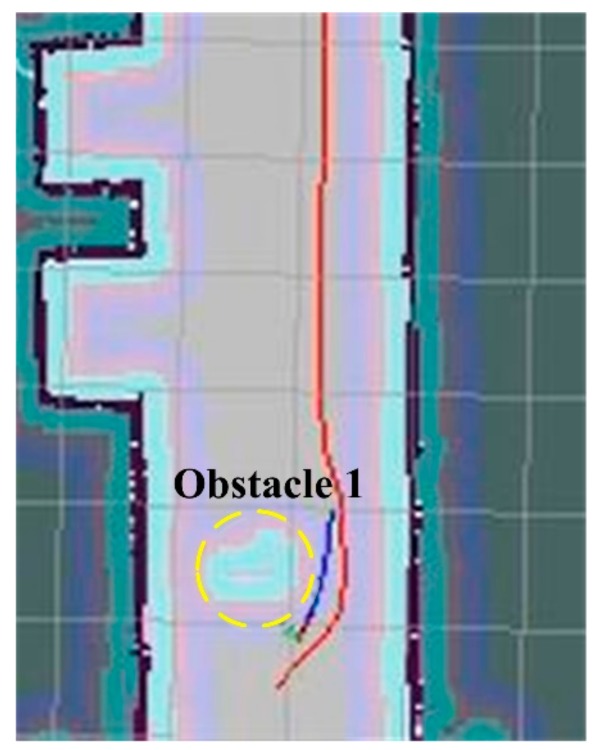
Obstacle avoidance during the test. The red and blue curves are the global and local planning paths based on A* and D* algorithms, respectively.

Experimental results can be seen in Figure 24. The robot can be accurately navigated to the target location, even though no information is available on the original map. During the transport, the robot can automatically turn on the D* algorithm to adjust the planned path to avoid the obstacles. In Figure 24, the area with the yellow circle is the obstacle. The red and blue curves are the global and local planning paths based on A* and D* algorithms, respectively. When the global and local paths are conflicting, the local path has priority. It was also noticed that the time needed to process D* algorithm is longer than that based on the A* algorithm when obstacles are shown in the planned path.

**Figure 25:**
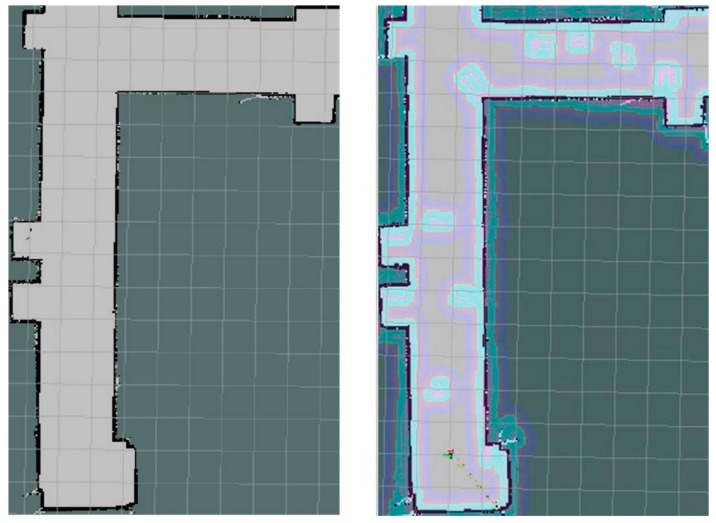
A comparison of the obtained map before (left) and after (right) the navigation.

The developed reconnaissance robot can dynamically update the map according to the surrounding environment. The completed map after the navigation is shown in Figure 25 (right), while Figure 25 (left) shows the map before the navigation. It can be seen that the information of the obstacles is added after the navigation, which can save a lot of time for the relevant post-processing.

## 5. A Comprehensive Test under Fire Conditions

**Figure 26:**
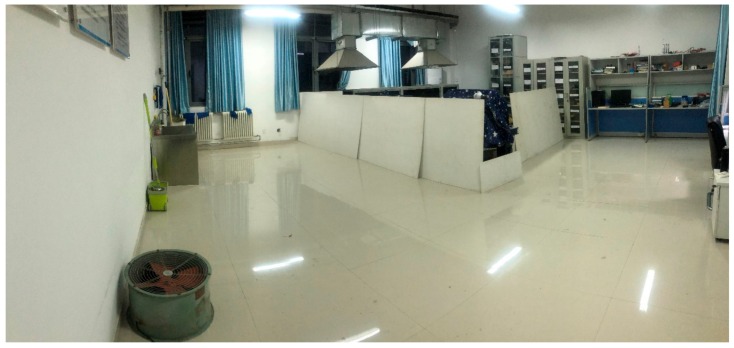
Simulated fire scenario inside an indoor environment.

To test the viability of the developed robot in this study, a comprehensive test was undertaken under fire conditions. To simulate the fire scenario, an indoor environment was selected, while fireproof panels were adopted to construct an L-shaped area. The constructed L-shaped area was about 50 m^2^, as shown in Figure 26.

**Figure 27:**
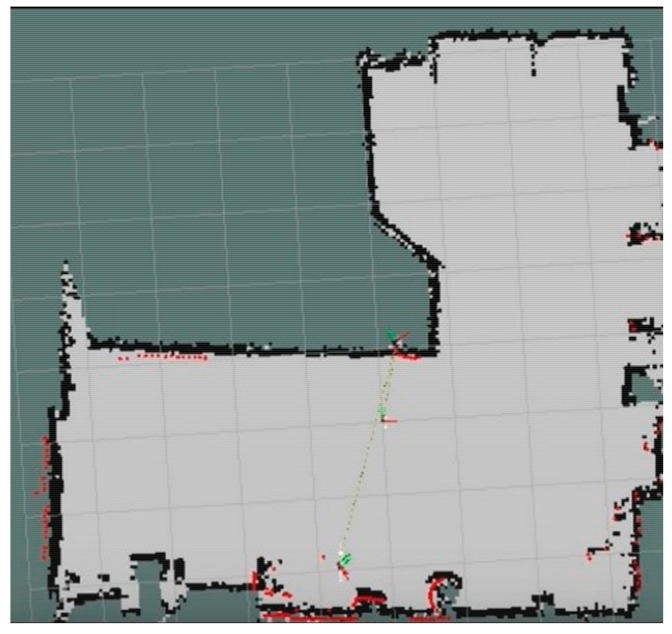
Constructed occupancy grid mapping for the simulated fire environment.

Before the experiment, the map of the experimental environment was constructed, as shown in Figure 27. Compared with Figure 26, it can be seen that the constructed map shows good reducibility when compared to the real situation.

**Figure 28:**
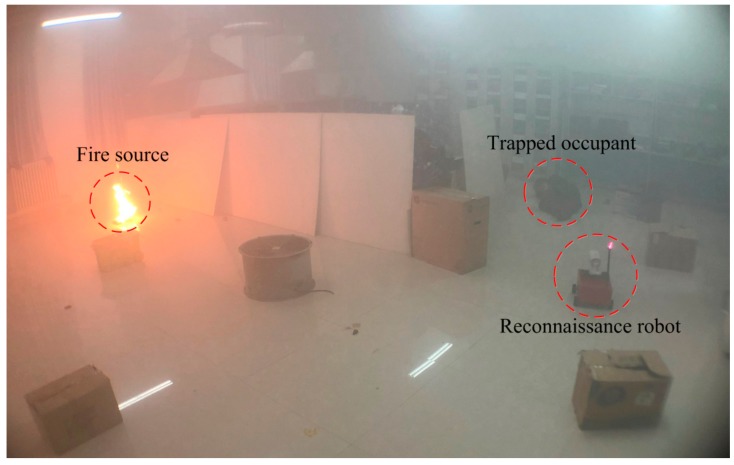
A simulated fire scenario with random obstacles, the fire source is on the left side of the L- shaped area, and a trapped occupant is on the right side of the L-shaped area. (This picture was taken at the beginning of the experiment, and the smoke completely covered the experimental environment after that).

During the experiment, to be more consistent with the real fire scenario, several obstacles were positioned randomly inside the tested environment to simulate collapsed walls or other structures under fire. The performance of obstacle avoidance for the developed fire reconnaissance robot can also be tested by these obstacles. A combined n-heptane pool fire and smoke cake with ammonium at the left end of the L-shaped area was adopted to simulate a fire scenario of both high temperature and dense smoke. A volunteer lay on the floor at the right end of the L-shaped area, which was to simulate a trapped occupant under fire conditions, as shown in Figure 28.

**Figure 29:**
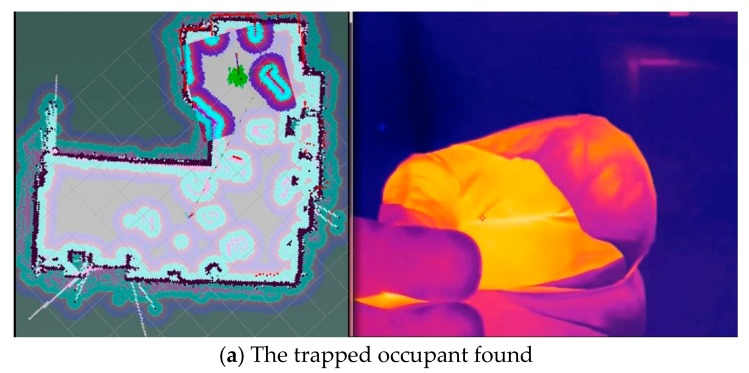

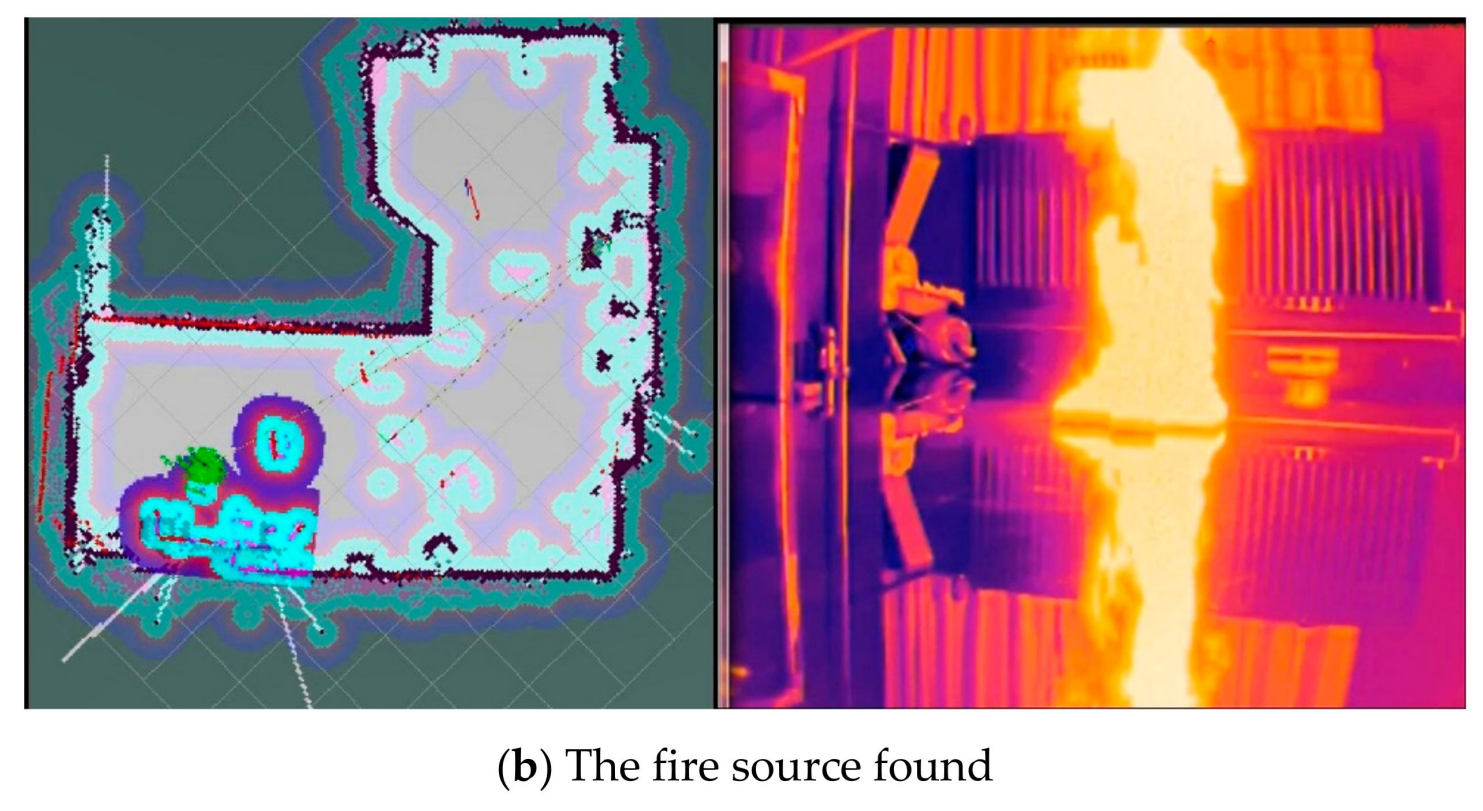
The results of the comprehensive test under fire conditions.

The developed fire reconnaissance robot was positioned inside the target area. The searching function of the robot was triggered and started to search for the trapped occupant and the fire source. The results after the search are shown in Figure 29. It can be seen from this figure that the video surveillance system clearly displays the information of both occupant and flame and the positions of the occupant and flame can be easily obtained by the robot, which is significant for the rescuing processes under fire conditions. Several areas in white can also be observed from the constructed map, which shows the position and size of the obstacles. It was indicated that the robot can easily avoid the obstacles and also update the constructed map according to the acquired obstacle information during the navigation.

## 6. Conclusions

A fire reconnaissance robot was developed in this study to benefit detection and rescuing processes under fire conditions. It adopts an infrared thermal image technology to detect the fire environment, uses SLAM technology to construct the real-time map, and utilizes A* and D* mixed algorithms for path planning and obstacle avoidance. The obtained information such as videos are transferred simultaneously to an AR goggle worn by the firefighters to ensure that they can focus on the rescue tasks by freeing their hands. To verify the performance of the developed robot, experimental tests were undertaken in a typical L-shaped space with both room and corridor. It was found that the robot showed a low mapping error of 3.43% and a high positioning accuracy of 0.31 m. A comprehensive test in a fire environment was simulated by an n-heptane pool fire together with an ammonium smoke cake, where the video surveillance system clearly displayed accurately the information of both trapped occupant and flame, and their positions could be easily obtained by the robot. The developed fire reconnaissance robot can enhance firefighters’ ability to understand the surrounding environment and improve their firefighting and rescuing efficiency while ensuring their safety under fire conditions.

In the next research, we will simulate a more realistic fire scenario, with a scenario testing for fire protection, system stability, and functional realization of the robot, together with attempts to increase robot data error correction capability testing, and exploring data fusion technology. 

## Figures and Tables

**Table 1:** Technical parameters of commonly adopted indoor positioning technology.

Technology	Positioning Accuracy	Measurement Principle	Require Base Station
UWB ^a^	Cm–m	Time from objective reflection	Yes
WLAN ^b^	m	Location fingerprinting	Yes
RFID ^c^	Mm–dm	Similarity detection and location fingerprinting	Yes
ZigBee	m	Location fingerprinting	Yes
Bluetooth	Dm–m	Location fingerprinting	Yes
SLAM ^d^	cm	Environmental feature extraction	No

^a^ UWB (Ultra Wide Band); ^b^ WLAN (Wireless Local Area Network); ^c^ RFID (radio frequency identification); ^d^ SLAM (simultaneous localization and mapping).

**Table 2:** The main technical parameters of the adopted LiDAR (RPLIDAR-A2).

Technical Parameter	Value	Maximum	Note
Measurement range	0.15–12 m	-	Measured based on white object with high reflectivity
Measurement resolution	<0.5 mm	N.A.	The measured object is within 1.5 m
Scanning angle	0–360°	N.A.	-
Angular resolution	0.9°	1.35°	Scanning frequency of 10 Hz

**Table 3:** A comparison of the positioning accuracy to the previous technologies.

Technology	Size of the Tested Space	Positioning Accuracy (m)	References
PDR ^a^	9.7 m × 5.94 m	1.96	Sun et al. [24]
UWB ^b^	6 m × 8 m	0.2	Segura et al. [25]
Camera/ PDR	16 m × 7.7 m	0.56	Zhou et al. [26]
GPS ^c^/UWB/ MARG ^d^	Business center	3.2	Zhang et al. [27]
Stereo Camera	8 m × 8.4 m × 4 m	0.677	Yang et al. [28]
BLE ^e^ fingerprint, fuzzy logic	52.5 m × 12.5 m	0.43	AL-Madani et al. [29]
SLAM ^f^	22.2 m × 2.7 m	0.31	This study

^a^ PDR (Pedestrian Dead Reckoning); ^b^ UWB (Ultra Wide Band); ^c^ GPS (Global Positioning System); ^d^ MARG (Magnetic Angular Rate and Gravity); ^e^ BLE(Bluetooth Low Energy); ^f^ SLAM (Simultaneous Localization And Mapping).

**Table 4:** The navigation errors.

Navigation Point	Navigation Distance (m)	Distance Error (m)	Distance Error Percentage (%)	Angle Error
1	12.0	0.26	2.17	4°
2	27.3	0.82	3.00	0°
3	36.2	1.26	3.48	10°
4	43.3	1.27	2.93	5°
